# Shearwave Elastography Increases Diagnostic Accuracy in Characterization of Breast Lesions

**DOI:** 10.1097/MD.0000000000003146

**Published:** 2016-03-25

**Authors:** Wei Lin Ng, Kartini Rahmat, Farhana Fadzli, Faizatul Izza Rozalli, Mohammad Nazri Mohd-Shah, Patricia Ann Chandran, Caroline Judy Westerhout, Anushya Vijayananthan, Yang Faridah Abdul Aziz

**Affiliations:** From the Department of Biomedical Imaging (WLN, KR, FF, FIR, MNM-S, CJW, AV, YFA), and Department of Pathology (PAC), Faculty of Medicine, University of Malaya, Kuala Lumpur, Malaysia.

## Abstract

The purpose of this study was to investigate the diagnostic efficacy of shearwave elastography (SWE) in differentiating between benign and malignant breast lesions.

One hundred and fifty-nine lesions were assessed using B-mode ultrasound (US) and SWE parameters were recorded (Emax, Emean, Emin, Eratio, SD). SWE measurements were then correlated with histopathological diagnosis.

The final sample contained 85 benign and 74 malignant lesions. The maximum stiffness (Emax) with a cutoff point of **≥** 56.0 kPa (based on ROC curves) provided sensitivity of 100.0%, specificity of 97.6%, positive predictive value (PPV) of 97.4%, and negative predictive value (NPV) of 100% in detecting malignant lesions. A cutoff of **≥**80 kPa managed to downgrade 95.5% of the Breast Imaging-Reporting and Data System (BI-RADS) 4a lesions to BI-RADS 3, negating the need for biopsy. Using a combination of BI-RADS and SWE, the authors managed to improve the PPV from 2.3% to 50% in BI-RADS 4a lesions.

SWE of the breast provides highly specific and sensitive quantitative values that are beneficial in the characterization of breast lesions. Our results showed that Emax is the most accurate value for differentiating benign from malignant lesions.

## INTRODUCTION

Breast ultrasound (US) is an indispensable tool in the investigation of breast pathology, especially for the characterization of mammographically or clinically detected lesions, supplementary evaluation in young women as well as in dense breasts, and the triple assessment test. Characterization of breast abnormalities with B-mode US is based on specific description criteria as defined by the American College of Radiology (ACR) in the Breast Imaging-Reporting and Data System (BI-RADS) lexicon.^1^ Recently, there has been great interest in the utilization of qualitative and quantitative information on breast lesions derived through breast elastography to distinguish between benign and malignant lesions. Currently, there exist several types of breast elastography, including strain imaging by compression, acoustic radiation force impulse (ARFI), and shearwave elastography (SWE). Strain elastography generates qualitative and semiquantitative breast tissue elasticity properties based on strain ratio and width ratio, whereas SWE produces additional quantitative values including Emax, Emean, Emin, and Eratio (in kilopascal [kPa] units).^2,3^

In shearwave imaging, an acoustic radiation force induces laterally moving shearwaves within the tissue in place of mechanical external compression. The shearwave speed is proportional to the square root of the tissue's shear modulus, which is approximately equal to one-third of Young modulus (assuming an isotrophic, pure elastic, homogeneous, and incompressible medium).^4^ Shearwaves are generated by displacement induced at the breast lesions which represents the viscoelastic properties of the tissue, thus producing a quantitative value.^5^ The velocity information can be mapped to create an image of the stiffness, with the option of measuring SWE features such as the minimum, mean, and maximum elasticity in a region of interest.^6^ These results are captured in real time.

As SWE reliably produces consistent images, it is particularly helpful in clinical situations where consistent image generation is critical, such as serial studies of masses. For example, SWE could provide crucial input in the follow-up of probably benign masses or the monitoring of breast carcinoma responses to neoadjuvant chemotherapy.

The objective of this study is to demonstrate that SWE is a specific and sensitive diagnostic tool for characterizing breast lesions that is capable of using both qualitative and quantitative data to assess different tissue stiffness specifically in BI-RADS 3 and 4 lesions, thereby reducing the number of unnecessary biopsies.

## METHODS AND MATERIALS

This study was conducted at the breast-imaging unit of the Biomedical Imaging department in University Malaya Medical Centre (UMMC) between June 2012 and April 2013. The study was approved by the institutional Medical Ethics Committee Board (Ethics approval no. 943.20). Informed consent was obtained from all patients before recruitment.

All subjects with breast lesions categorized as BIRADS 4 and above who were scheduled for US core biopsy and/or surgical biopsy were included in this study. We also included several subjects with BIRADS 2 and 3 lesions who were scheduled for biopsy, either upon patient request or due to other risk factors. The subjects were either referred from the breast assessment clinic with palpable lumps (105 lesions) or had sonographically detected lesions (54 lesions). Patients without conclusive histological diagnosis and those with a previously known histological diagnosis were excluded.

**FIGURE 1:**
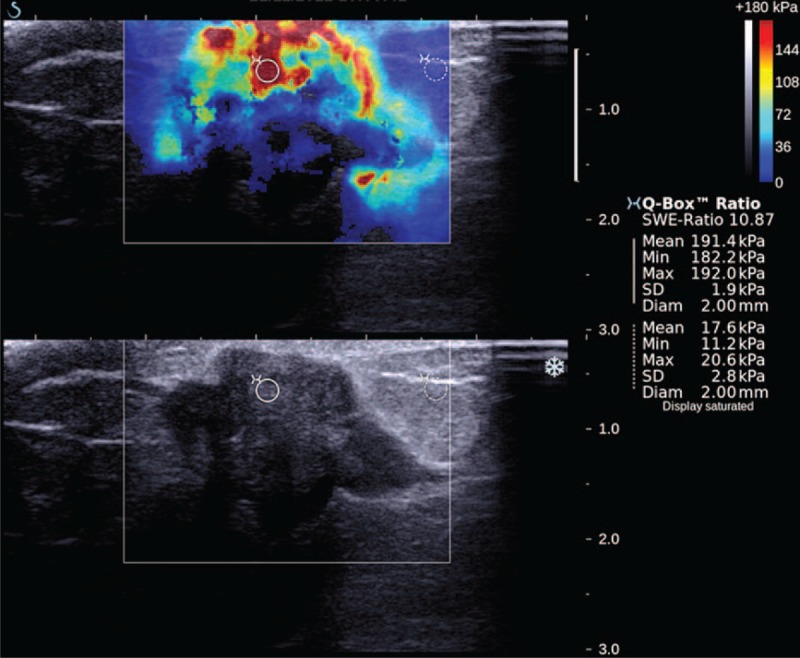
Quantitative measurements: A circular ROI of 2-mm diameter was placed at the area of maximum stiffness of the lesion with another ROI placed at the normal breast tissue at the same depth. Emean is the mean elasticity of the lesion, Emin is the minimum elasticity of the lesion, Emax is the maximum elasticity of the lesion, and SD is the standard deviation of the results. SWE ratio is the ratio of the lesion to the normal tissue. Qualitative measurements: Shape, homogeneity of lesion.

All scans were performed using the Aixplorer ultrasound system (SuperSonic Imagine, Aix en Provence, France) using a 15-4 MHz linear transducer probe. Two radiologists specializing in breast imaging were responsible for scanning the patients (FF and KR with 4 and 10 years of experience, respectively). Both radiologists were blinded to the histological diagnosis. The study protocol was formulated and standardized after performing an initial pilot study in accordance to previous literature.^6–10^ The US examination initially produced Standard B-mode gray scale images, and generated elastography images after an extra 3 to 5 minutes. A 3 × 3 cm color map in transverse plane was placed on the region encompassing the lesion and its immediate surrounding tissues. The probe was held stationary with no added pressure over the region of interest (ROI) during the SWE measurements. A circular ROI with a diameter of 2 mm was placed at the center of the lesion to attain a color map. The 2-mm diameter was chosen in line with other recently published studies.^9,10^ Real-time shearwave images were captured after viewing the color map. The color map provided a visual representation of tissue stiffness, with different shades of a spectrum of colors: blue for softer tissues and red for harder tissues. The ROI's SWE readings consisted of maximum elasticity (Emax), mean elasticity (Emean), minimum elasticity (Emin), and ratio of lesion to surrounding tissue (Eratio), with their standard deviations (SD). The color map was also represented numerically with lower values corresponding to softer tissues, that is, blue, and higher values to harder tissues, that is, red (Figure 1). The range of the color scale was 0 to 180 kPa. The lower limit of the chromatic scale was adjusted to 180 kPa to display evident differences in tissue stiffness based on the color map; this setting is regarded as the calibrated optimum scale for breast study as per the manufacturer's recommendation. However, the quantitative measurement scale was set at a maximum of 300 kPa.

The breast lesions were also qualitatively analyzed and scored using the ACR BI-RADS classification system. Grading of the BI-RADS score based on B-mode gray-scale images was conducted by the 2 breast radiologists (FF and KR), who were blinded to the patients’ physical examination, mammography, and histopathology diagnosis. As all of the cases were either indeterminate or suspicious on US, the final assessment categories were BI-RADS 3 (probably benign), BI-RADS 4 (suspicious), and BI-RADS 5 (highly suggestive of malignancy). We further subcategorized suspicious lesions into BI-RADS 4a (low risk of malignancy), 4b (intermediate risk of malignancy), and 4c (high risk of malignancy).

Statistical analysis was then performed using SPSS version 20.0 (SPSS Inc, Chicago, IL). Each of the quantitative SWE parameters (Emax, Emean, Emin, and Eratio) and qualitative lesion characteristics of the lesions was analyzed using receiver-operating characteristic (ROC) curves. BI-RADS category 3 was considered as negative for malignancy, whereas categories 4a and above were considered positive. The quantitative lesion characteristics were then correlated with the criterion standard of diagnosis—the histopathological results. The optimal cutoff values of the SWE parameters in predicting malignancy were calculated based on ROC curves, and the sensitivity, specificity, positive predictive value (PPV), negative predictive value (NPV), and accuracy were determined. The value of *P* < 0.05 was considered statistically significant. The interobserver agreement of SWE measurements was assessed using interclass correlation coefficients (ICCs).

## RESULTS

A total of 159 lesions in 152 patients were examined during the study period. The patients’ age ranged between 11 and 93 years with a mean age of 52 years. The majority of patients scanned were Chinese (42%), followed by Malays (37%), Indians (16%), and other ethnicities (5%).

Seventy-four (46.5%) lesions were malignant, whereas 85 (53.5%) were benign. The majority of the malignant lesions were infiltrating ductal carcinomas (IDCs), whereas the majority of the benign lesions were fibroadenomas. Further breakdown of the histopathology is listed in Table 1. The sizes of the malignant lesions ranged from 0.5 to 9.0 cm (mean ± SD: 2.1 ± 1.2 cm), whereas the sizes of the benign lesions ranged from 0.3 to 5.0 cm (mean ± SD: 1.4 ± 1.0 cm).

**TABLE 1:**
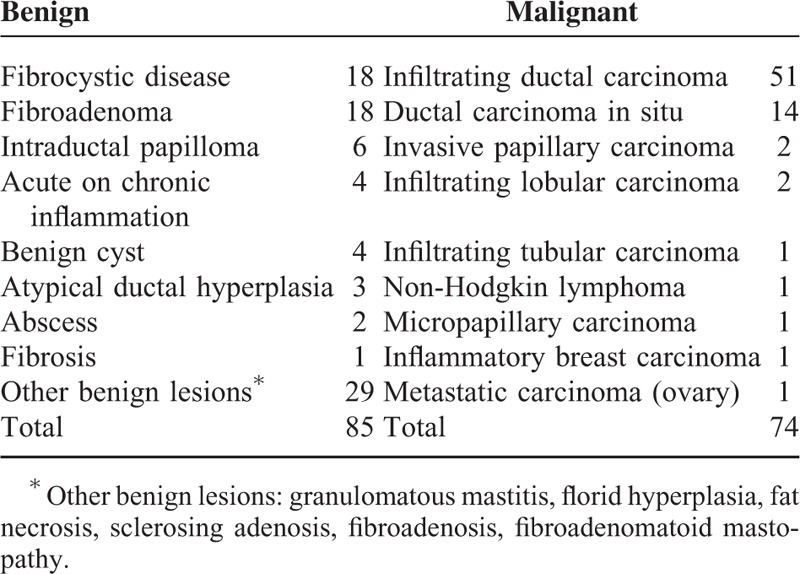
Histopathological Results of 159 Breast Lesions

### Gray Scale Ultrasound Findings

Of the 159 lesions, 26 (16.4%) were classified as BI-RADS 3, 80 (50.4%) as BI-RADS 4, and 53 (33.2%) as BI-RADS 5. Further division into BI-RADS 4a, 4b, and 4c was also performed according to the ACR BI-RADS^1,11^ (see Table 2). When using BI-RADS 4 and 5 as a predictor of malignancy, conventional US showed a sensitivity of 100%, specificity of 30.6%, PPV of 55.6%, and a NPV of 100%. The PPV of gray scale US in detecting malignancy increased gradually as the BI-RADS category increased: 0.0% (n = 26) for BI-RADS 3, 2.3% (n = 43) for BI-RADS 4a, 31.3% (n = 16) for BI-RADS 4b, 75% (n = 20) for BI-RADS 4c, and 100% (n = 53) for BI-RADS 5.

**TABLE 2:**
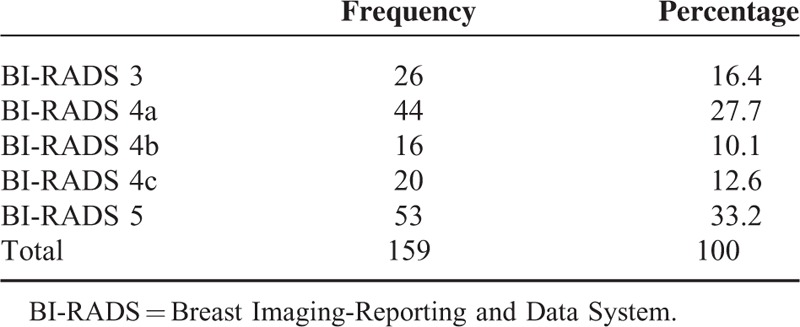
Number of Lesions According to BI-RADS Classification

### Quantitative SWE Findings

**FIGURE 2:**
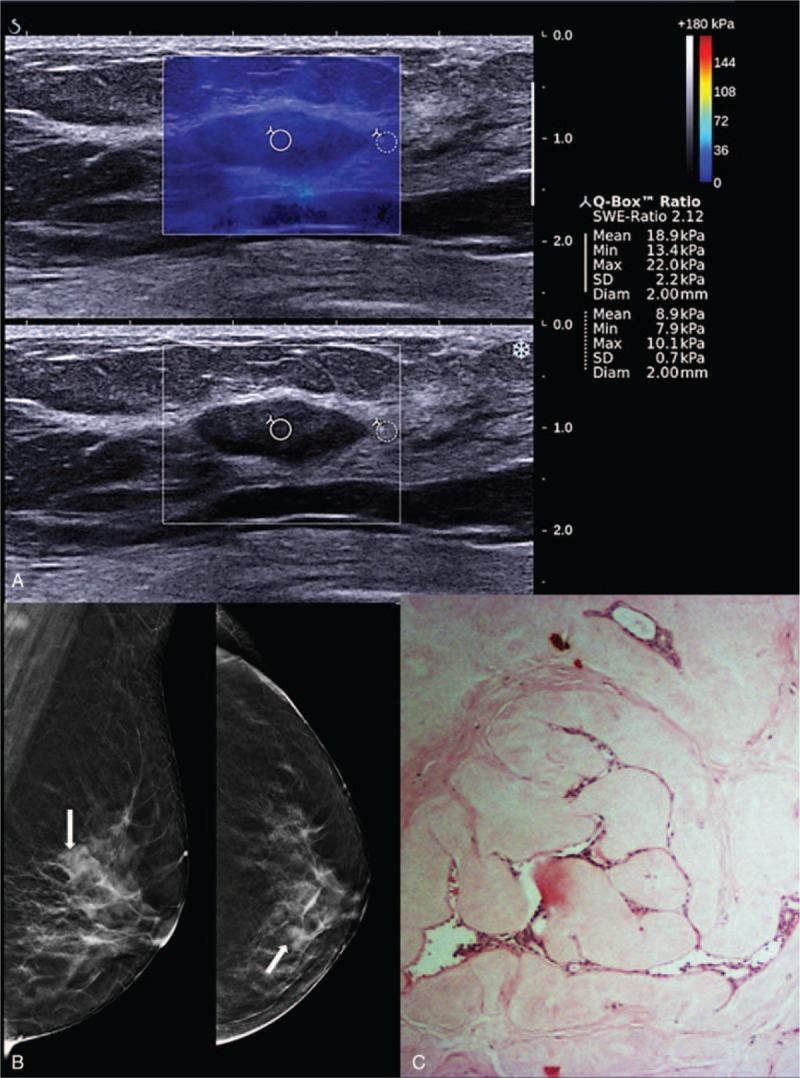
Shearwave elastography (SWE) of a Breast Imaging-Reporting and Data System (BI-RADS) 3 lesion in a 51-year-old woman with a palpable lump. (A) Gray scale B-mode ultrasound showed a well-defined oval hypoechoic lesion. Shearwave imaging showed a homogenous blue color map (soft) with Emax of 22.0 kPa. (B) Digital breast tomosynthesis (DBT) in MLO and CC views with white arrows showing a partially circumscribed lesion at the left upper inner quadrant. (C) Photomicrograph (H and E, ×40) of a surgical excision specimen of a fibroadenoma.

**FIGURE 3:**
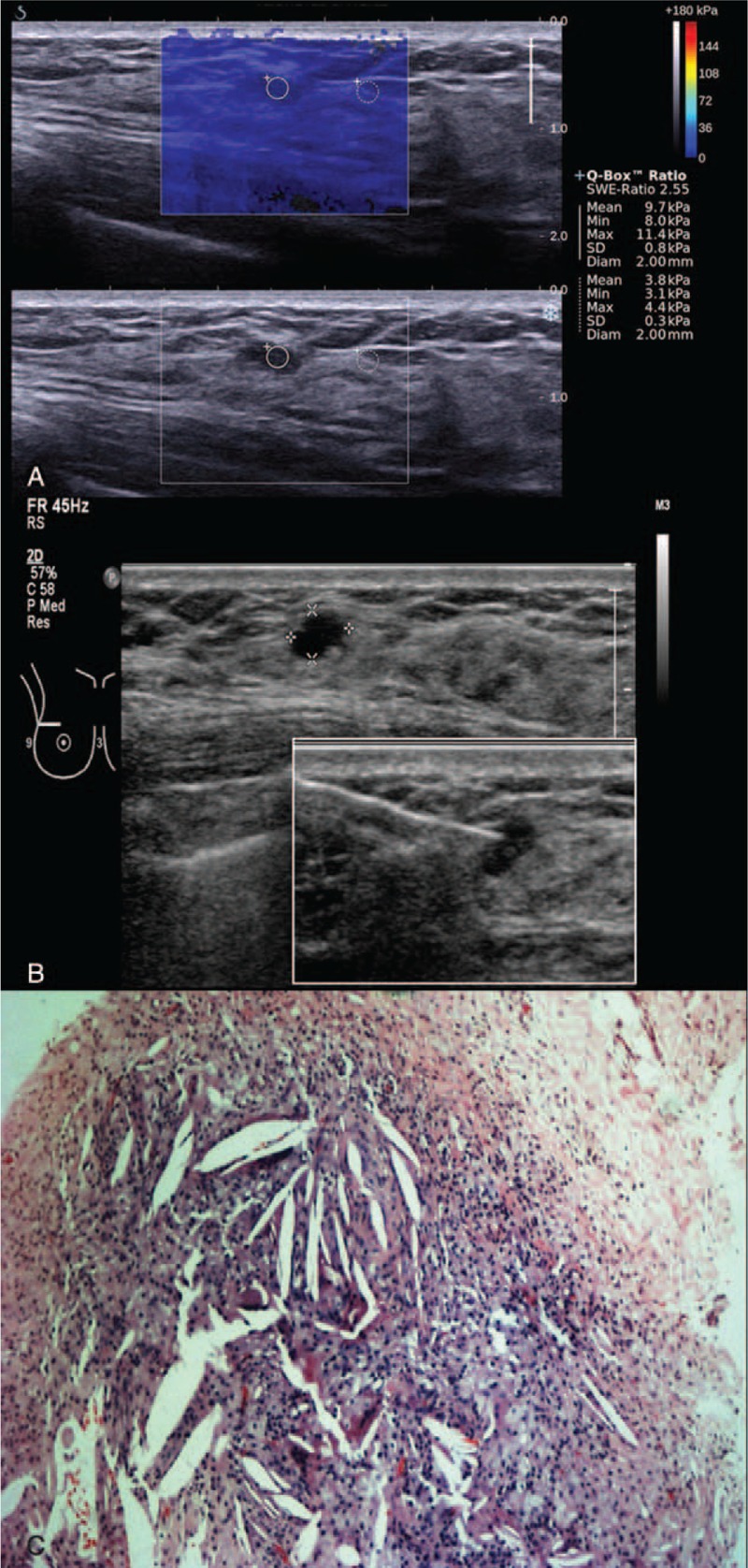
Shearwave elastography (SWE) of a Breast Imaging-Reporting and Data System (BI-RADS) 3 lesion in a 49-year-old woman. (A) Shearwave imaging showed a homogenous color map (soft) with Emax of 11.4 kPa. (B) Gray scale B-mode ultrasound showing a well-defined oval hypoechoic lesion. Photo insert shows an image guide core needle biopsy of lesion. (C) Right breast digital mammogram in MLO and CC views showing a benign focus of macrocalcification. No dominant mass or suspicious cluster of microcalcifications. (D) Photomicrograph (H and E, ×40) of core biopsy of a fat necrosis.

**FIGURE 4:**
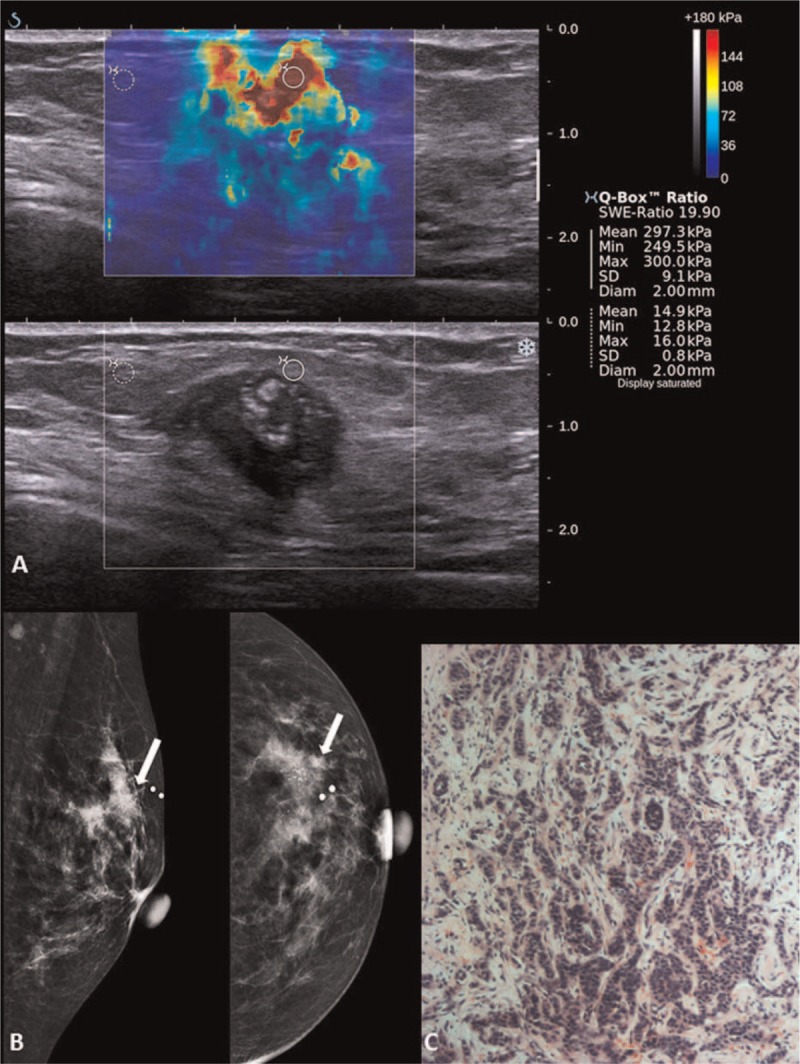
Shearwave elastography (SWE) of a Breast Imaging-Reporting and Data System (BI-RADS) 5 lesion in a 73-year-old woman with a palpable lump. (A) Gray scale B-mode ultrasound showed an irregular hypoechoic lesion with posterior shadowing and internal calcifications. Shearwave imaging showed a heterogeneous color map and Emax of 300 kPa. (B) Left breast digital mammogram in MLO and CC views with arrows showing a high-density spiculated lesion with clusters of microcalcifications at the left upper outer quadrant. (C) Photomicrograph (H and E, ×40) of a surgical excision specimen of an infiltrating lobular carcinoma.

**FIGURE 5:**
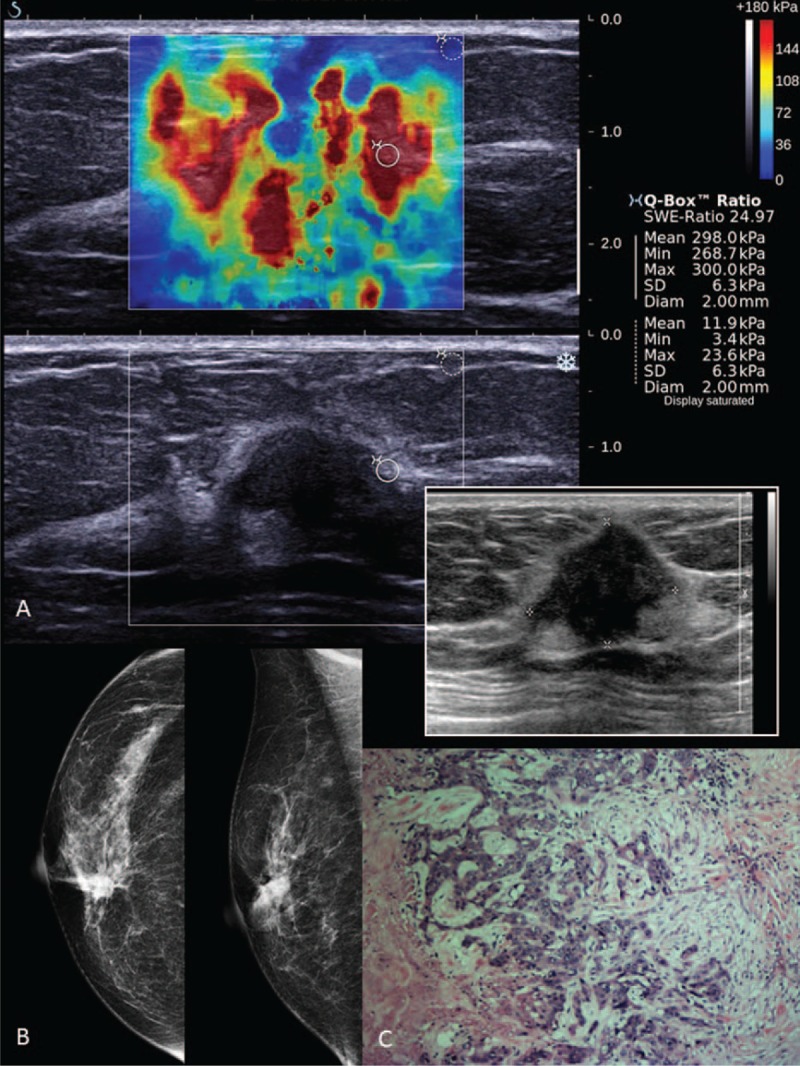
Shearwave elastography (SWE) of a Breast Imaging-Reporting and Data System (BI-RADS) 5 lesion in a 65-year-old woman with a palpable lump. (A) Shearwave imaging showed a heterogeneous color map and Emax of 300 kPa. Photo insert shows a gray scale B-mode ultrasound of an irregular lobulated hypoechoic lesion (taller than wider). (B) Right breast digital mammograms in MLO and CC views showing a high-density spiculated lesion with associated microcalcifications at the right upper inner quadrant. (C) Photomicrograph (H and E, ×40) of surgical excision specimen of an infiltrating ductal carcinoma.

Benign lesions generally showed a uniform homogeneity on the color map with no difference in lesion color map size compared to B-mode. Examples of benign lesions are shown in Figures 2 and 3, whereas malignant lesions are shown in Figures 4 and 5.

**FIGURE 6:**
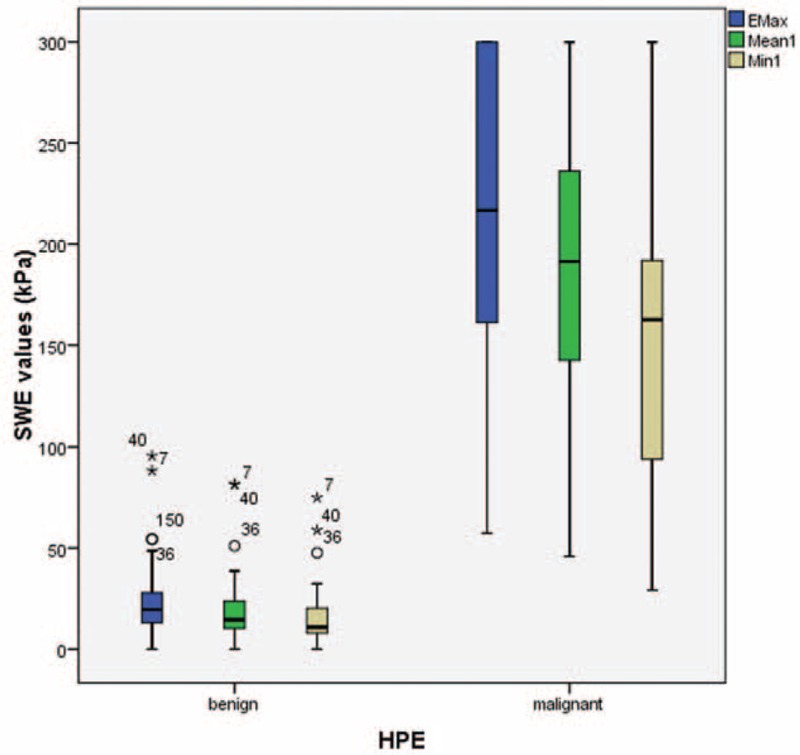
Box plot chart of shearwave elastography (SWE) parameters of benign and malignant breast lesions. According to the Mann–Whitney test, *P* values are statistically significant between malignant and benign lesions (*P* = 0.001).

**FIGURE 7:**
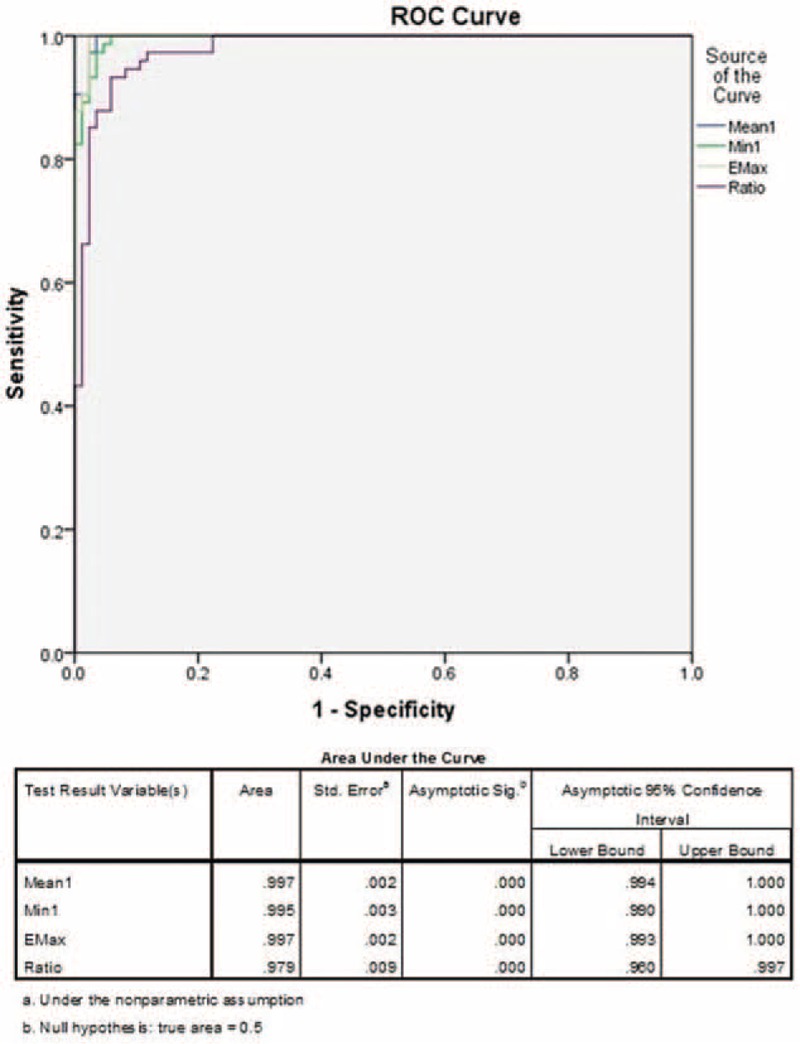
The receiver-operating characteristics (ROC) curves of Emax, Emean, and Eratio shearwave elastography (SWE) measurements in detecting malignant breast lesions. The area under the curves (AUCs) for Emax, Emean, Emin, and Eratio were 0.997, 0.995, 0.997, and 0.979, respectively. Cutoff values deduced from the ROC curves were Emax ≥56 kPa, Emean ≥42 kPa, Emin ≥29 kPa, and ratio ≥2.2.

The mean values of Emax, Emean, Emin, Eratio, and Standard Deviation were statistically significantly higher in malignant lesions (Table 3, Figure 6). Using these quantitative values, the cutoff points for each parameter were deduced using ROC curves. The areas under the curves (AUCs) were 0.997 for maximum elasticity (Emax), 0.997 for mean elasticity (Emean), and 0.979 for mass/fat elasticity ratio (Eratio) (Figure 7). The optimal cutoff points of Emax, Emean, Emin, and Ratio (independent of B-mode BI-RADS category) were ≥56 kPa, ≥42 kPa, ≥29 kPa, and 2.2, respectively. Emax was the most sensitive (100%) and specific (97.6%) in detecting malignant breast lesions (see Table 4). The results of SWE measurements alone superseded B-mode BI-RADS classification in detecting malignant lesions.

**TABLE 3:**
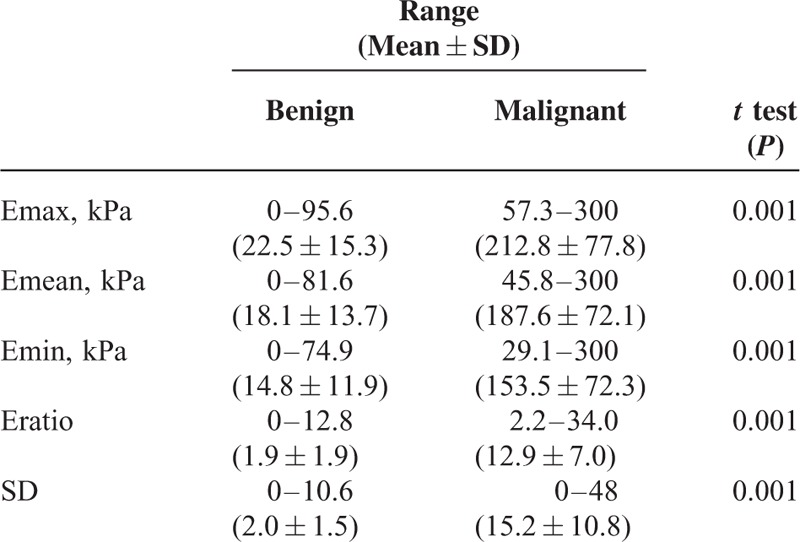
SWE Parameters Between Benign and Malignant Lesions

**TABLE 4:**
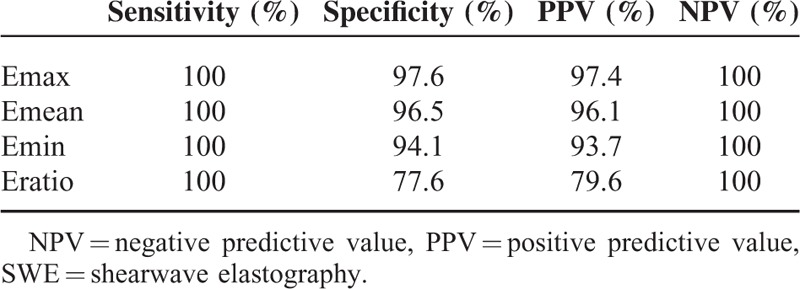
Sensitivity, Specificity, PPV, and NPV of SWE to Detect Malignant Breast Lesions Using Emax ≥56, Emean ≥42, Emin ≥29, Ratio ≥2.2

### Combination of Conventional Ultrasound and SWE Findings

We examined the feasibility of applying SWE measurements to potentially downgrade BI-RADS 4a lesions to BI-RADS 3, negating the need for biopsy. Using an Emax ROC, a range of cutoff points (40, 50, 60, 70, and 80 kPa) was tested, and showed identical accuracies. We chose an Emax cutoff of ≥80 kPa (based on our data that found no BI-RADS 3 lesion to be > 54.4kPa), which showed 100% sensitivity, 97.7% specificity, 50% PPV and 100% NPV. Using this technique, we were able to downgrade 42 lesions out of 44 from BI-RADS 4a to BI-RADS 3. Of the remaining 2 lesions, one was proven to be malignant.

Given that the PPV of BI-RADS 4b lesions was low (31.2%), an Emax cutoff of 80 kPa was then applied, also yielding promising results (100% sensitivity, 90.9% specificity, 83.3% PPV and 100% NPV). In BI-RADS 4c lesions, the PPV further improved from 75% to 100% (93.3% sensitivity, 100% specificity, 100% PPV, and 83.3% NPV).

The Emean, Emin, and Emax values for normal tissue and lesions, as well as the Eratio values showed very strong interobserver agreement (ICC > 0.9). The consistency of agreement between the 2 observers was slightly better for lesion SWE measurements (95% CI: 0.950, 0.998) than for normal tissue (95% CI:0.643, 0.997).

### Infiltrating Ductal Carcinoma Findings

**FIGURE 8:**
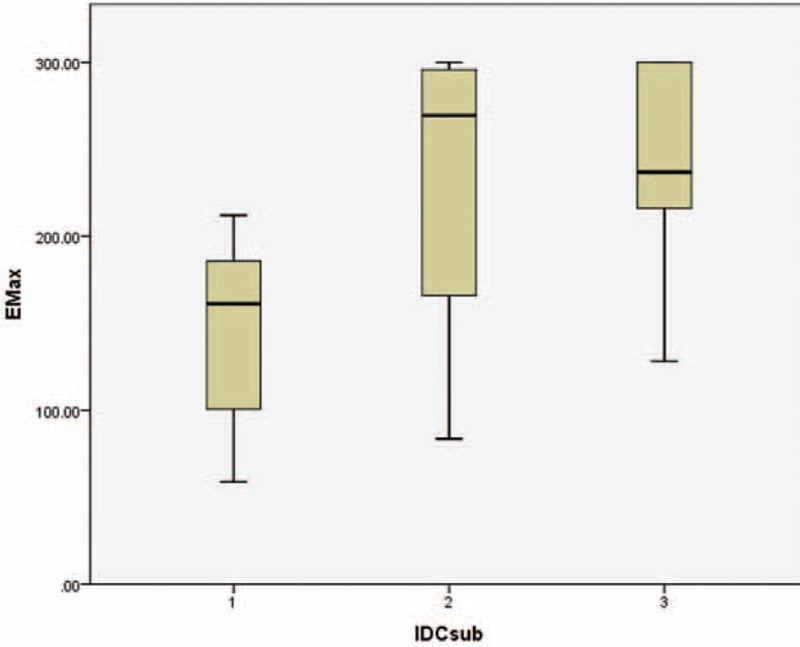
Boxplot showing the distributions of Emax values across different grades of infiltrating ductal carcinoma (IDC) lesions, demonstrating a gradual increase in Emax values as the IDC grades increased. Kuskal-Wallis test *P* value 0.022.

**FIGURE 9:**
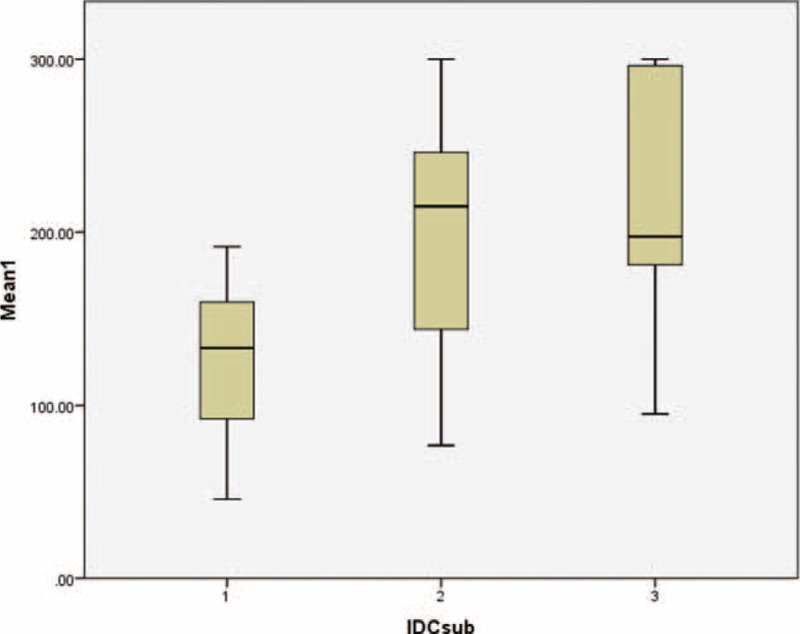
Boxplot showing the distributions of Emean values across different grades of infiltrating ductal carcinoma (IDC) lesions, demonstrating a gradual increase in Emean values as the IDC grades increased. Kuskal-Wallis test *P* value 0.031.

Forty-five of 51 IDC cases (6 results were not available) were graded based on Bloom-Richardson's grading system. Of these 45 cases, 17.6% were grade 1, 45.1% were grade 2, and 25.5% were grade 3. Using Kruskal-Wallis tests for independent samples, we were able to determine that there was a significant difference in the distributions of Emax and Emean values between the different IDC grades, with *P* values of 0.022 and 0.031, respectively. The results also reported a trend wherein the higher IDC grades tended to have higher SWE values (Figures 8 and 9).

## DISCUSSION

Previous studies using shearwave imaging have demonstrated the clinical usefulness of elasticity values in differentiating benign and malignant solid lesions, by quantitatively establishing the increased stiffness of the latter masses.^8,9,11,12^ Benign lesions tended to be stiffer or harder than normal breast tissue but softer than malignant lesions.

In this study, we found a benign preponderance and low PPV in subjects categorized as BI-RADS 4, suggesting that the number of benign cases undergoing biopsy could be safely reduced and instead followed-up over short-term intervals. Also, according to ACR 2013, only 3% to 10% of BI-RADS 4a lesions undergoing biopsy are malignant. Previous studies by Berg et al, Chang et al, Athanasious et al, and Lee et al^8–10,13^ have additionally found that unnecessary biopsies can be reduced by downgrading BI-RADS 4a lesions to BI-RADS 3.

In our study, BI-RADS 3 lesions were either biopsied or excised because of various reasons, such as strong family history of breast carcinoma, high risk factors, or as per patient/surgeon request. In a previous study by Hong et al,^14^ the PPV and NPV obtained for malignant lesion detection ranged from 62% to 86% and 78% to 90%, respectively, when using BI-RADS grading alone, which is based on conventional gray scale sonographic characteristics. To obviate the need for biopsy, Stavros et al^11^ also produced a classification scheme utilizing ultrasonography, and found the PPV and NPV to range from 48.2% to 91.8% and 86.9% to 96.5%. In our study, the use of conventional US alone in the diagnosis of BI-RADS 4/5 lesions produced a moderate PPV of 55.6%, but a NPV of 100%. This shows that lack of suspicious characteristics helped exclude malignancy among breast lesions. However, the presence of indeterminate or suspicious morphologic features resulted in unnecessary biopsies or diagnostic surgical procedures in approximately 50% of our benign patients.

**FIGURE 10:**
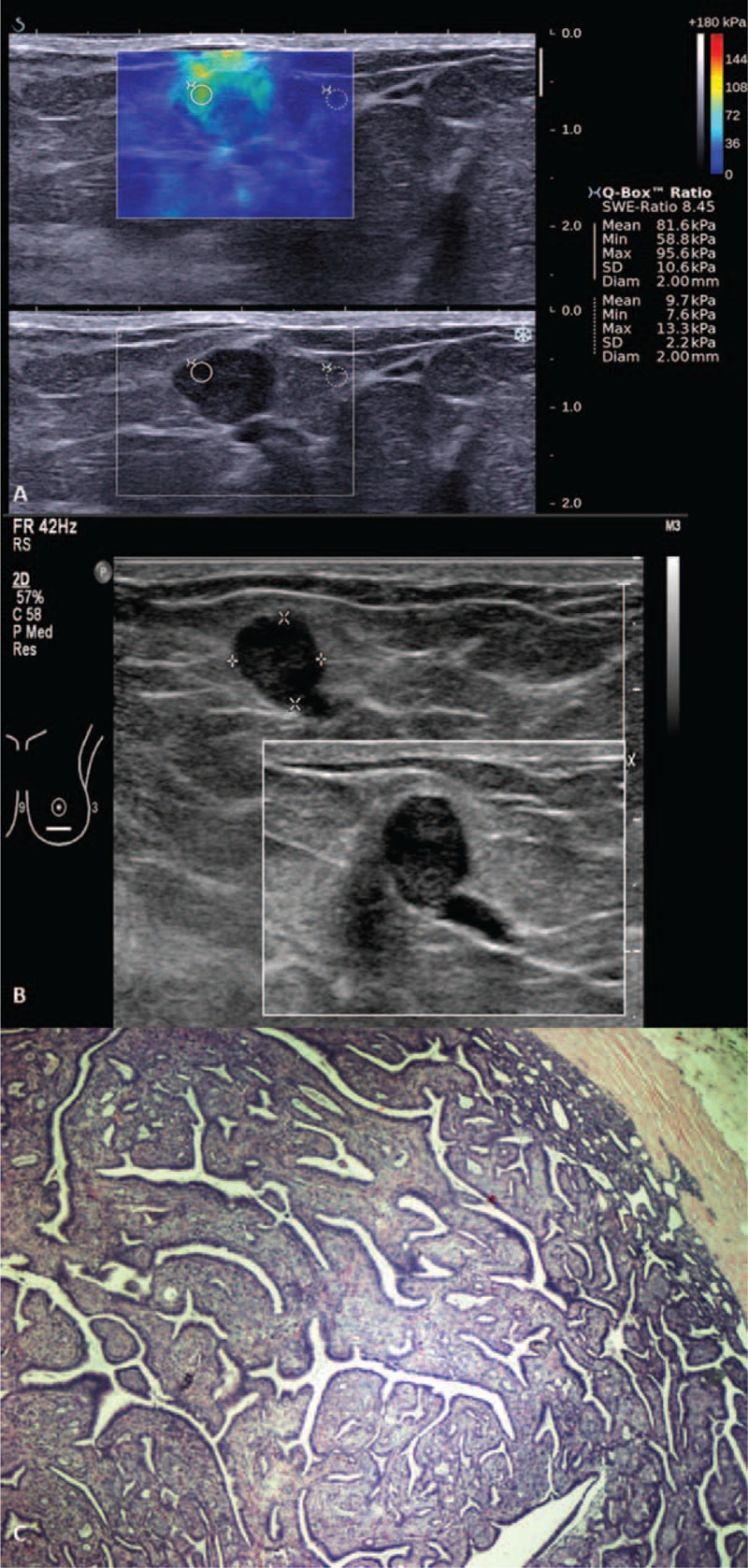
Shearwave elastography (SWE) of a Breast Imaging-Reporting and Data System (BI-RADS) 4a lesion in a 27-year-old woman with a palpable lump and nipple discharge. (A) Shearwave imaging showed a heterogeneous color map and Emax of 95.6 kPa. (B) Gray scale ultrasound showed a lobulated hypoechoic lesion continuous with a dilated duct. (C) Photomicrograph (H and E, ×40) of surgical excision specimen of an intraductal papilloma.

All lesions in the BI-RADS 3 category had a NPV of 100% based on both gray-scale US and when used in combination with SWE, using a cutoff point of Emax ≥56 kPa. However, in BI-RADS 4a category lesions, the PPV was 2.3% for conventional US alone. This percentage increased to 50% after applying a combination of B-mode imaging with SWE, using a cutoff point of ≥80 kPa. Two lesions in BI-RADS 4a showed ≥80 kPa on SWE. These 2 lesions were an intraductal papilloma with an Emax of 95.6kPa (false-positive) (Figure 10) and an infiltrating ductal carcinoma with an Emax of 275.30 kPa (true positive).

In addition to conducting sonographic shearwave evaluation of the breast lesions, we attempted to determine the cutoff values to improve the specificity of lesion characterization using various SWE parameters (Emean, Emax, or Eratio). Specifically, this could be achieved by either downgrading lesions from BIRADS 4 to BI-RADS 3 or upgrading lesions from BI-RADS 3 to BI-RADS 4a. Table 5 shows a comparison of our results to that of other studies in terms of parameter choice for upgrading BI-RADS 3 to BI-RADS 4. Based on the table, it is evident that the cutoff value is variable.

**TABLE 5:**
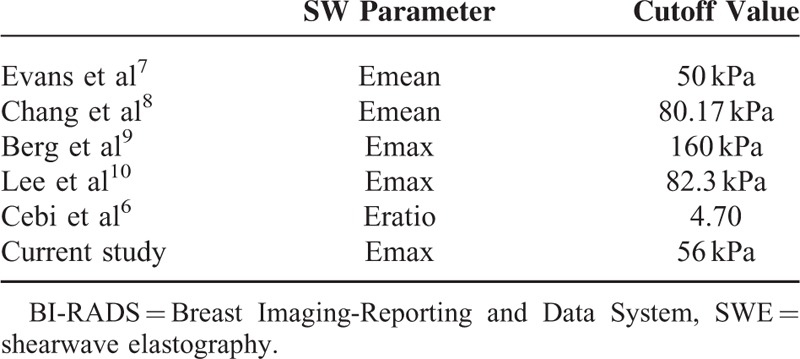
Comparison of Cutoff Points for SW Elastography Parameter, for Upgrading BI-RADS 3 to BI-RADS 4 (Selected By Each Study as the Preferred Parameter) From Recent Studies

Two of the 5 studies in the literature used Emax, whereas the other 3 used Eratio and Emean values. The cutoff Emax value we used to upgrade our lesions was 56 kPa, which is lower than that used by Berg et al (160 kPa) and Lee et al (82.3 kPa), respectively.^9,10^ Berg et al^9^ also calculated the Emax for downgrading BI-RADS 4a lesions, and identified 2 values of ≥30 kPa and ≥80 kPa, the former being a more conservative value. We chose a cutoff value of ≥80 kPa for our studied population, which was similar to the “aggressive” cutoff found in the study done by Berg et al. Another study by Athanasiou et al^13^ used Emean to recode BI-RADS to 0, −1, or +1 to determine whether the BI-RADS should be upgraded or downgraded. However in our study, we achieved optimal combination of sensitivity, specificity, PPV, and NPV with Emax values.

The degree of stiffness of the lesions also correlates with the level of aggression in breast cancer.^15^ Based on Bloom and Richardson's histological grading of infiltrating ductal carcinoma, we found a correlation between increased tumor stiffness and higher tumor grade.^16^ Our findings were similar to previous studies by Cebi et al and Evans et al,^6,7^ wherein malignant lesions of higher grades tended to have higher SWE values (both Emean and Emax). It has been hypothesized that a rise in cellularity owing to augmented mitosis contributes to the increased stiffness of the lesion.^17^ For example, Lee^18^ et al investigated the histopathological characteristics of invasive breast carcinoma and found that SWE values were affected by fibrosis, particularly tumor cellularity and necrosis. Similarly, a recent study conducted by Matsubayashi et al^19^ found malignant breast lesions to show a higher degree of fibrosis as compared to benign lesions, which correlates to MRI elastography readings. Finally, Vinnicombe et al^20^ who studied a total of 320 malignant lesions also found a false-negative value of 7.3% and concluded that soft cancers were likely to be low-grade and small, and that pure DCIS were more likely to display benign shearwave features.

In general, breast SWE has been shown to be highly reproducible in the assessment of both intraobserver and interobserver variability.^21^ In our study, we also concluded that the interobserver variability between our 2 readers was strong (ICC > 0.9). Lesions, compared with normal surrounding tissues, showed a slightly better interobserver agreement, likely owing to the fact that discrete lesions are easier to consistently assess between different observers. Qualitative parameters, including color overlay and pattern classification, were also found in our study to be useful in characterizing breast lesions as shown by Gweon et al.^22^

One of the limitations of our study is the relatively small sample size of malignant lesions. However, the percentage of malignant lesions was relatively high (46.5%). This could be attributed to the fact that our center is a tertiary referral unit and thus our patient population was made up of a larger percentage of suspicious lesions, which had been previously screened by other centers. A second factor could be related to the opportunistic screening practiced in our country rather than the use of an established population based screening programme. Further clinical studies with multicenter collaborations may provide more confirmatory evidence regarding appropriate cutoff values for clinical use and generate a better yield of histopathological results.

## CONCLUSION

Our study shows that SWE improves sensitivity, specificity, PPV, NPV, and accuracy in characterization of benign and malignant lesions on US. An Emax value of ≥80 kPa managed to further refine our BI-RADS 4 category, whereas an Emax of ≥56 kPa was most useful in predicting the outcome of biopsies. Owing to the promising results of SWE, we highly recommend that it should be incorporated into routine clinical practice, particularly in situations of greater clinical value, that is, when deciding whether indeterminate lesions should undergo biopsy or just close surveillance. The data from our study adds to the current body of literature by providing more evidence for SWE utilization, as advocated in the recent 2013 ACR BI-RADS Atlas.^1^
